# Relation between the Disability of the Arm, Shoulder and Hand Score and Muscle Strength in Post-Cardiac Surgery Patients

**DOI:** 10.3390/diseases5040031

**Published:** 2017-11-27

**Authors:** Kazuhiro P. Izawa, Yusuke Kasahara, Koji Hiraki, Yasuyuki Hirano, Satoshi Watanabe

**Affiliations:** 1 Graduate School of Health Sciences, Kobe University, 7-10-2 Tomogaoka, Suna-ku, Kobe 654-0142, Japan; izawapk@ga2.so-net.ne.jp; 2 Department of Rehabilitation Medicine, St. Marianna University School of Medicine Yokohama-city Seibu Hospital, 1197-1, yasashi-cho, asahi-ku, Yokohama 241-0811, Japan; kasahara.y@marianna-u.ac.jp; 3 Department of Rehabilitation Medicine, St. Marianna University School of Medicine Hospital, 2-16-1 Sugao, Miyamae-ku, Kawasaki 216-8511, Japan; hiraki7@marianna-u.ac.jp; 4 Department of Physical Therapy, Tokushima Bunri University, 180, Nishihama, Yamashiro-cho, Tokushima 770-8514, Japan; hirano@tks.bunri-u.ac.jp

**Keywords:** cardiac surgery, rehabilitation, handgrip strength, knee extensor muscle strength, DASH

## Abstract

**Background:** The Disabilities of the Arm, Shoulder, and Hand (DASH) questionnaire is a valid and reliable patient-reported outcome measure. DASH can be assessed by self-reported upper extremity disability and symptoms. We aimed to examine the relationship between the physiological outcome of muscle strength and the DASH score after cardiac surgery. **Methods:** This cross-sectional study assessed 50 consecutive cardiac patients that were undergoing cardiac surgery. Physiological outcomes of handgrip strength and knee extensor muscle strength and the DASH score were measured at one month after cardiac surgery and were assessed. Results were analyzed using Spearman correlation coefficients. **Results:** The final analysis comprised 43 patients (men: 32, women: 11; age: 62.1 ± 9.1 years; body mass index: 22.1 ± 4.7 kg/m^2^; left ventricular ejection fraction: 53.5 ± 13.7%). Respective handgrip strength, knee extensor muscle strength, and DASH score were 27.4 ± 8.3 kgf, 1.6 ± 0.4 Nm/kg, and 13.3 ± 12.3, respectively. The DASH score correlated negatively with handgrip strength (*r* = −0.38, *p* = 0.01) and with knee extensor muscle strength (*r* = −0.32, *p* = 0.04). **Conclusion:** Physiological outcomes of both handgrip strength and knee extensor muscle strength correlated negatively with the DASH score. The DASH score appears to be a valuable tool with which to assess cardiac patients with poor physiological outcomes, particularly handgrip strength as a measure of upper extremity function, which is probably easier to follow over time than lower extremity function after patients complete cardiac rehabilitation.

## 1. Introduction

The reported goals of cardiac rehabilitation (CR) for post-cardiac surgery patients have been to improve exercise capacity and physiological outcomes of muscle strength, reduce coronary risk factors, improve health-related quality of life, and reduce subsequent cardiac events, sudden death, all-cause mortality, and hospitalization costs [1,2,3,4,5,6].

Of these, the physiological outcomes of handgrip strength and knee extensor muscle strength of post-cardiac surgery patients who underwent coronary artery bypass grafting and valve replacement have also been reported, and the findings have supported the improvements that are attained with CR [3,7]. However, both handgrip strength and knee extensor muscle strength in such patients were lower than those in patients who underwent percutaneous coronary intervention (PCI) performed one month after the onset of acute myocardial infarction or after cardiac surgery [7].

With regard to muscle proteolysis in cardiac surgery patients, a recent study suggested that muscle proteolysis due to postoperative hypercatabolism is responsible for the functional decline that is observed in patients undergoing cardiac surgery [8]. Differences in hypercatabolism between patients undergoing PCI and cardiac surgery may be one of the reasons for differences in the physiological outcomes of handgrip strength and knee extensor muscle strength.

In contrast, in regard to patient-reported outcomes of CR, we previously reported that upper- and lower-body self-efficacy for physical activity and physical component summary and mental component summary scores, as assessed by Short Form-36 in cardiac surgery patients were also lower than those of patients undergoing PCI performed for acute myocardial infarction one month after the onset of infarction or after cardiac surgery [7].

One of the patient-reported outcome measures is the Disability of the Arm, Shoulder and Hand (DASH) questionnaire, which is an upper extremity-specific outcome measure [9,10].

The rationale behind the use of one outcome measure for different upper extremity disorders is that the upper extremities are a functional unit [11,12]. In this respect, the DASH questionnaire would be suitable because it is mainly a measure of disability [11,12].

The DASH score is based on a 30-item, patient-reported outcomes questionnaire designed to measure physical function and symptoms in patients with musculoskeletal disorders of the upper limbs. The items are based on the degree of difficulty in performing different physical activities with the problem arm, shoulder, or hand; the severity of each of the symptoms of pain, activity-related pain, tingling, weakness, and stiffness; and the effect of the problem on social activities, work, sleep, and self-image [11,12]. Each item has five response options [9,10].

A previous report suggested that postoperative muscle weakness was associated with interleukin (IL)-6 production as an index of the site of inflammation immediately after cardiac surgery [13]. In addition, post-cardiac surgery patients need to follow sternal precautions to protect their sternum after open-heart surgery [14]. Muscle weakness may result from both the limitation of upper extremity movement and infection in these patients. Therefore, upper extremity muscle strength may limit patients’ activities of daily living. Moreover, the items in the DASH questionnaire to no small extent include not only upper extremity function but also its relation to lower extremity function [9,10].

Thus, we hypothesized that there would be a correlation between physiological outcomes of handgrip strength and knee extensor muscle strength and the DASH score. However, due to the current lack of evidence, it is unknown whether physiological outcomes of both handgrip strength and knee extensor muscle strength correlate directly with the DASH outcome measures in post-surgery cardiac patients. Therefore, the purpose of the present study was to assess the correlation between physiological outcomes of handgrip strength and knee extensor muscle strength, and the DASH score in post-cardiac surgery patients.

## 2. Methods

### 2.1. Participants

The present cross-sectional study comprised 50 consecutive cardiac patients who had undergone coronary artery bypass grafting or valve replacement and visited hospital as outpatients one month after cardiac surgery, and who were referred to the Department of Rehabilitation Medicine for first-time evaluation of physiological outcomes and DASH score. Exclusion criteria included patients with neurological, peripheral vascular, orthopedic, pulmonary, and advanced renal disease.

Medical records review was undertaken to evaluate patient characteristics that included age, sex, body mass index, left ventricular ejection fraction, etiology, co-morbidity, and medications. We evaluated these characteristics one month after cardiac surgery. At this same time, physiological outcomes were assessed and the DASH score was calculated.

The present study complied with the principles of the Declaration of Helsinki regarding investigations in humans, and it was approved by the local institutional review board of our hospital. Informed consent was obtained from each patient.

### 2.2. Physiological Outcomes

We assessed handgrip strength and knee extensor muscle strength as measures of physiological outcomes [2,3,7]. A standard adjustable-handle JAMAR dynamometer (Bissell Healthcare Co., Grand Rapids, MI, USA) was used for the measurement of handgrip strength as an index of upper limb muscle power, and was set at the second grip position for all of the subjects [2,3,7]. Attention was paid to a possible Valsalva effect, and three measurements each were made on both hands. After the measurements, we calculated the average of the highest value of the right- plus left-hand grip strength/2 and used the highest value measured as the index of hand grip strength.

The Biodex System 2 isokinetic dynamometer (Biodex Medical Systems, Inc., New York, NY, USA) was used to measure knee extensor muscle strength as the index of lower limb muscle strength. Testing was performed for a maximum of five repetitions of knee extension at isokinetic speeds of 60°/s. Isokinetic test results were analyzed with the Biodex System 2 software [2,3,7]. We measured the knee extension muscular strength peak torque per body weight value of both knees. Thereafter, we calculated the average of the highest value of the right- plus left-side knee extension muscular strength/2, and used the maximum value that was obtained as the index of knee extension muscular strength [2,3,7].

### 2.3. DASH Questionnaire

We used the Japanese version of the DASH questionnaire [9,10,11,12], for which, reliability, validity, and responsiveness in Japanese patients were assessed previously [9,10]. The DASH is a 30-item disability/symptom scale examining the patient’s health status during the preceding week [9,10,11,12]. The items measure the degree of difficulty in performing different physical activities with the problem arm, shoulder, or hand; the severity of each of the symptoms of pain, activity-related pain, tingling, weakness, and stiffness; and the problem’s impact on social activities, work, sleep, and self-image. Each item has five response options. To arrive at the DASH score, the scores for all of the items are then used to calculate a scale score ranging from 0 (no disability) to 100 (most severe disability) [9,10,11,12].

### 2.4. Statistical Analysis

Results related to clinical characteristics of the patients are expressed as mean ± standard deviation (SD) and percent. Spearman’s rank correlation coefficient analysis was used to test the relations between the physiological outcome variables of handgrip strength and DASH scores and knee extensor muscle strength and DASH scores. A *p* value of <0.05 was considered to indicate statistical significance. Statistical analyses were performed with IBM SPSS 22.0 J statistical software (IBM SPSS Japan, Inc., Tokyo, Japan).

## 3. Results

### 3.1. Clinical Characteristics of the Patients

**Figure 1:**
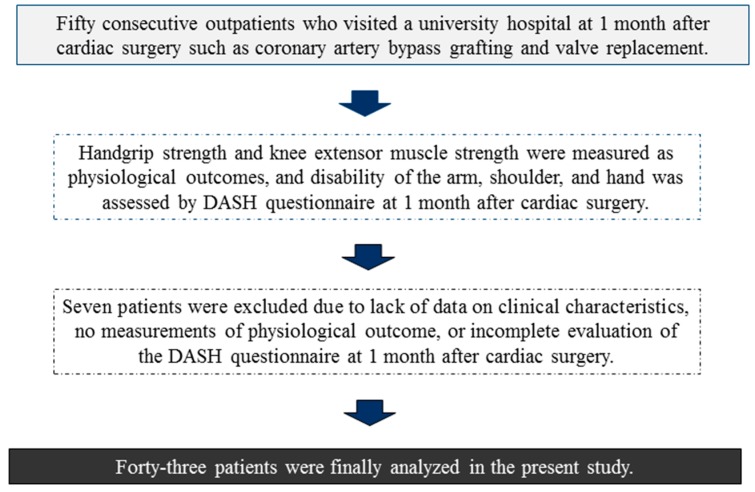
Patient flow during the present study.

Patient flow during the present study is presented in Figure 1. Of the 50 patients, seven were excluded from the study because of lack of data on clinical characteristics, no measurement of physiological outcomes, or incomplete evaluation of the DASH questionnaire at one month after cardiac surgery. Thus, the remaining 43 patients were analyzed in the present study. Patient characteristics are presented in Table 1.

### 3.2. Handgrip Strength, Knee Extensor Muscle Strength, and DASH Scores

Handgrip strength, knee extensor muscle strength, and DASH scores of the patients are presented in Table 2. Average handgrip strength, knee extensor muscle strength, and DASH scores were 27.4 ± 8.3 kgf, 1.6 ± 0.4 Nm/kg, and 13.3 ± 12.3, respectively.

### 3.3. Relation between Physiological Outcomes of Handgrip and Knee Extensor Muscle Strength and DASH Score

**Figure 2:**
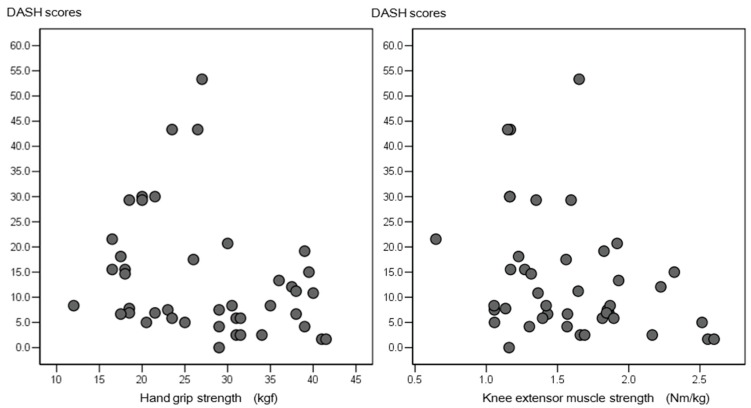
Relationship between handgrip strength, knee extensor muscle strength, and DASH scores of the patients. DASH, Disabilities of the Arm, Shoulder, and Hand.

Negative correlations were found between the DASH score and both handgrip strength (*r* = −0.38, *p* = 0.01) and knee extensor muscle strength (*r* = −0.32, *p* = 0.04) (Figure 2).

## 4. Discussion

This is first time, to our knowledge, that disabilities of the arm, shoulder, and hand, as assessed with the DASH scores of Japanese post-cardiac surgery patients, has been evaluated in relation to physiological outcomes of muscle strength. The main findings of this study are that the physiological outcomes of both handgrip strength and knee extensor muscle strength correlated negatively with the values of the DASH score in the patients at 1 month after cardiac surgery.

Previous studies suggested that the deterioration of skeletal muscle due to surgery, which occurs rapidly during the postoperative period, is a morbidity with serious complications [13,15,16]. In the present study, the average values of handgrip strength and knee extensor muscle strength of the patients were 27.4 ± 8.3 kgf and 1.6 ± 0.4 Nm/kg, respectively.

Myers et al. [17] suggested that there are age-adjusted relative risks of death for each of the major risk factors in male subjects who achieve a peak exercise capacity of <5 METs (metabolic equivalent of task), as compared with the fittest subjects and cardiac patients. Another previous study showed that an exercise capacity of ≥5 METs in male heart failure patients was equivalent to an approximate handgrip strength 35.2 kgf and knee extensor muscle strength of 1.70 Nm/kg [18].

The average values of handgrip strength (27.4 kgf) and knee extensor muscle strength (1.6 Nm/kg) in the present study were inferior to the above target variables that were related to an exercise capacity of five METs. However, there are also differences of handgrip strength and knee extensor muscle strength between men and women [19]. In the present study, 74.4% (32/43) of the patients were men, so we need to evaluate sex difference more equally in future trials.

Several previous reports suggested a positive correlation between knee extensor muscle strength and gait speed. When knee extensor muscle strength was <1.20 Nm/kg, the correlation between these values was even stronger, and gait speed was very slow in elderly patients [18,20]. In terms of gait speed, the average knee extensor muscle strength of 1.6 Nm/kg in the present study was superior to the target variable of 1.20 Nm/kg.

The DASH score in the present study correlated negatively, although weakly so, with handgrip strength. There could be several reasons for these correlations in the post-cardiac surgery patients. After cardiac surgery, medical staff may require their patients to follow sternal precautions, such as a method to protect their sternum after open-heart surgery [14]. Such precautions are used in almost all patients after this surgery to avoid dehiscence of the sternum as it is healing [14], and are meant to protect the patient and prevent possible infection.

In addition, previous reports suggested that postoperative muscle weakness was associated with IL-6 production immediately after cardiac surgery [8,13]. Reasons for postoperative muscle weakness may not only be limited activity, but also to infection following cardiac surgery. We did not evaluate biomarkers such as IL-6 as an index of the site of inflammation, so the possible relationship between muscle strength and biomarkers is unknown, and we would to include this in future studies. Muscle weakness may result from both the limitations of upper extremity activity and infection. These may be reasons for the negative correlation of DASH scores with upper extremity strength in the present study. Thus, the present results may support the targeting of upper extremity variables related to activities of daily living in cardiac surgery patients.

However, in the present study there was also a weak negative correlation between knee extensor muscle strength and the DASH score. Although we could not determine the reason for this, the DASH questionnaire items to no small extent included not only upper extremity function, but also references to lower extremity function as well, such as ‘put your luggage on the overhead racks’, ‘does gardening’, and ‘carried some heavy stuff’ [9,10]. Therefore, knee extensor muscle strength may have some relation to the DASH score in the results of the present study. The DASH score could possibly be used not only as an indicator of improvement in upper extremity strength, but also that in lower extremity strength in cardiac surgery patients.

Limitations of the present study include its small sample size, of which almost three-quarters were men. This study also included younger and older cardiac surgery patients. Additional analysis of sex or age or etiology or co-morbidities-related differences in DASH score in cardiac patients is also required. We also did not evaluate the effects of postoperative complications or the starting day of the earlier phase I CR program on selection bias or of longer hospital stays. Data on DASH scores in relation to cardiac-related mortality or re-hospitalization were not assessed due to the limited amount of related data that was available. A previous study suggested that the DASH score correlated negatively with the physical, psychological, and environmental WHOQOL-BREF domains in patients adhesive capsulitis [21]. We also did not evaluate the relationship between other self-reported outcomes, nor do we know the longitudinal changes of these values in cardiac patients. Thus, we need to address these deficiencies in future longitudinal studies.

## 5. Conclusions

This study attempted to examine the relation between the DASH score and physiological outcomes of muscle strength after cardiac surgery. As a result, physiological outcomes of both handgrip strength and knee extensor muscle strength correlated negatively with the DASH score. This score might be a valuable tool with which to assess cardiac patients with poor physiological outcomes, particularly in terms of handgrip strength as a measure of upper extremity function, which is probably easier to follow over time than lower extremity strength after patients complete CR. This study lacks long-term follow-up data on the DASH score; therefore, additional study will be required to evaluate long-term outcomes. 

## Figures and Tables

**Table 1:** Clinical characteristics of the patients.

No. of Patients	43
Age (years, range)	62.1 ± 9.1, 42–78
Sex (male)	32
BMI (kg/m^2^)	22.1 ± 0.7
LVEF (%)	53.5 ± 0.7
Etiology (%)	
CABG	58.1
VR	41.9
Co-morbidity (%)	
Diabetes	37.8
Medications (%)	
Beta-blockers	47.7
ACEI/ARB	43.2
Diuretic	70.5

BMI, body mass index; LVEF, left ventricular ejection fraction; CABG, coronary artery bypass grafting; VR, valve replacement; ACEI, angiotensin converting enzyme inhibitor; ARB, angiotensin receptor blocker.

**Table 2:** Handgrip strength, knee extensor muscle strength, and Disabilities of the Arm, Shoulder, and Hand (DASH) scores of the patients.

Values	Average	Minimum	Maximum
Handgrip strength (kgf)	27.4 ± 8.3	12.0	42.0
Knee extensor muscle strength (Nm/kg)	1.6 ± 0.4	0.6	2.6
DASH score	13.3 ± 12.3	0.0	53.3

DASH, Disabilities of the Arm, Shoulder, and Hand.
